# Life satisfaction of patients in forensic psychiatry departments in the context of illness acceptance and clinical and demographic factors

**DOI:** 10.3389/fpsyt.2026.1996731

**Published:** 2026-10-28

**Authors:** Joanna Fojcik, Daniel Szawarnoga, Michał Górski

**Affiliations:** Department of Psychiatry, Department of Neurology, Faculty of Health Sciences in Katowice, Medical University of Silesia in Katowice, Katowice, Poland

**Keywords:** forensic psychiatry, isolation, life satisfaction, psychological well-being, SWLS

## Abstract

**Aim:**

The aim of the study was to assess the level of life satisfaction of forensic psychiatry patients and to identify selected demographic and clinical factors related to life satisfaction, with particular emphasis on illness acceptance.

**Material and methods:**

The study included 121 patients hospitalized in four forensic psychiatry departments. Life satisfaction was assessed using the Satisfaction with Life Scale (SWLS), and illness acceptance using the Acceptance of Illness Scale (AIS). The relationship between SWLS scores and age and AIS scores was analyzed, as well as differences in SWLS scores based on gender, duration of hospitalization, psychiatric diagnosis, and pharmacotherapy regimen. Spearman’s rank correlation, Student’s t-test, one-way ANOVA, and the Mann-Whitney U test were used depending on the type of variables. A nominal significance level of α = 0.05 was adopted. No p-value correction was applied due to multiple testing.

**Results:**

The mean SWLS score was 19.82 ± 7.50 points. The study group showed varied levels of life satisfaction. After converting the results to the sten scale, 39.7% of participants achieved results corresponding to a low level of life satisfaction, 26.4% - an average level, and 33.9% - a high level. The mean AIS score was 28.06 ± 7.70 points; low level of illness acceptance was found in 10.7% of participants, average in 47.1%, and high in 42.1%. There were no statistically significant differences in SWLS scores depending on gender (p = 0.412), psychiatric diagnosis (p = 0.951), pharmacotherapy regimen (p = 0.930), or duration of hospitalization (p = 0.211). There was no statistically significant correlation between the SWLS score and age (rho Spearman’s ratio = -0.166; p = 0.068). However, a weak positive correlation was observed between the AIS score and the SWLS score (rho Spearman’s α = 0.215; p = 0.018), statistically significant at the nominal level of α = 0.05.

**Conclusions:**

of life satisfaction were found among the patients included in the study. The largest group consisted of participants classified as low (39.7%), but the differences in numbers between categories did not indicate a clear dominance of one category over the other. In the study group, the most common levels of illness acceptance were medium (47.1%) or high (42.1%). There was no statistically significant correlation between SWLS scores and age, nor were there any statistically significant differences in SWLS scores based on gender, psychiatric diagnosis, pharmacotherapy regimen, or length of hospitalization. However, a weak positive correlation was observed between illness acceptance and life satisfaction, significant at the nominal level of α = 0.05. The cross-sectional nature of the study does not allow for conclusions about a causal relationship between these variables. The results suggest the validity of considering illness acceptance in further studies on life satisfaction in forensic psychiatric patients.

## 1 Entry

Life satisfaction is considered one of the basic components of subjective mental well-being and is an important indicator of quality of life, independent of objective health status or social status (1, 2). Mental health research increasingly emphasizes that subjective life assessment provides information complementary to classic symptomatic and functional measures, and that low levels of life satisfaction are associated with poorer prognosis, reduced therapeutic adherence, and a higher risk of rehospitalization (3–5).

Patients hospitalized in forensic psychiatry units constitute a population particularly vulnerable to reduced subjective well-being. Their functioning is simultaneously affected by severe mental disorders, prolonged hospitalization, compulsory treatment, and legal constraints (6, 7). Research indicates that a sense of lack of control, limited autonomy, and uncertainty regarding the duration of hospitalization are key factors reducing the quality of life in this group of patients (8, 9).

At the same time, the literature highlights the heterogeneity of the forensic psychiatric patient population. Some patients, despite long-term hospitalization, report moderate or high levels of life satisfaction, which is associated with the presence of personal resources, social support, and the quality of the therapeutic relationship (10–12).

One of the most commonly used tools for measuring global life satisfaction is the Satisfaction with Life Scale (SWLS), which has good psychometric properties and is widely used in clinical trials (1, 13).

## 2 Material and methods

### 2.1 Characteristics of the study group

The study covered patients hospitalized under security measures ordered under applicable legal provisions in the Polish forensic psychiatry system.

The study was conducted in four forensic psychiatry departments with a basic level of security. A total of 121 patients were enrolled in the study, including 30 patients from three centers and 31 patients from the fourth center. All centers operated within the framework of applicable Polish regulations regarding the implementation of security measures and basic security requirements. A common legal framework defines the basic principles of supervision, safety, and the organization of care. No separate statistical analysis of the results was conducted by center.

Participant recruitment took place between July and August 2025. The qualification process identified a total of 150 patients who met the study inclusion criteria. Of these, 29 (19.3%) did not consent to participate in the project. Ultimately, 121 individuals (N = 121) participated in the study. Reasons for refusal were not analyzed. This approach was intended to ensure voluntary participation and avoid imposing additional burdens on patients related to research procedures.

The average age of the respondents was 41 years (min. 19, max. 74). Men predominated in the group (79%). The most common marital status was single (64%), and the majority of respondents lived with their families (60%). The largest group consisted of people with secondary and vocational education, and the dominant form of professional activity was receiving a disability pension.

The largest group of patients in terms of length of stay in the ward were those hospitalized for less than a year (43% - 52). The next largest category was those who stayed in the ward for 1 to 3 years (30% - 36). Stays lasting 3 to 5 years occurred in 13% (16) of patients. 9% (11) of the study participants stayed in the ward for 5 to 9 years. The smallest group consisted of patients hospitalized for more than 9 years (5% - 6). For the analysis of SWLS results, the original five categories of hospitalization duration were combined into three groups: less than a year (n = 52), 1 to 3 years (n = 36), and more than 3 years (n = 33). The first two categories were retained, while the last three were combined due to their small numbers. The categories were combined into three groups to reduce the problem of small numbers in individual subgroups. This procedure allowed for comparisons between groups, but it was associated with the loss of some information resulting from the original differences in stay duration.

Information regarding psychiatric diagnosis was obtained for 116 patients, but this data was missing for 5 participants. Paranoid schizophrenia was recorded in 80 individuals, and other psychiatric diagnoses in 36 individuals. These constituted 66% and 30% of the total sample (N = 121), respectively. The “Other diagnoses” category included patients diagnosed with depression, anxiety disorders, substance use disorders, and intellectual disability. Due to the small size of each subgroup, these diagnoses were combined into a single category for statistical analyses. Data regarding diagnosis were collected using a demographic and clinical questionnaire.

In the analyzed group of patients, various pharmacological treatment regimens were used, including both monotherapies and multi-component therapies.

Typical pharmacotherapy listed in the study inclusion criteria is a criterion for qualifying patients for the study, while the subsequent refusal to take medications is an element of the actual therapeutic procedure.

Four categories were identified for the pharmacotherapy characteristics. Eighty-five patients were classified as receiving no mood stabilizer, while 31 were classified as receiving a mood stabilizer in combination with antipsychotic therapy. Additionally, two patients were receiving only a mood stabilizer, and three patients were not receiving pharmacotherapy. The comparison of SWLS scores by pharmacotherapy regimen included the first two groups. Patients receiving only a mood stabilizer (n = 2) and those without pharmacotherapy (n = 3) were not included in this comparison. Data on pharmacotherapy were obtained from the patients’ medical records.

The characteristics of the study group are presented in **Table 1**.

**Table 1:** Characteristics of the study group (N = 121).

Variable	Category/measure	Result
Age (years)	Mean ± SD	40.78 ± 11.55
	Median (Q1; Q3)	39 (32; 46)
	Min-max	19-74
Sex	Women	25 (20.7%)
	Men	96 (79.3%)
Length of stay in the ward	Less than a year	52 (43.0%)
	From 1 to 3 years	36 (29.8%)
	From 3 to 5 years	16 (13.2%)
	From 5 to 9 years old	11 (9.1%)
	Over 9 years old	6 (5.0%)
Psychiatric diagnosis	Paranoid schizophrenia	80 (66.1%)
	Other diagnoses	36 (29.8%)
	No data on diagnosis	5 (4.1%)
Drug therapy regimen	No mood stabilizer	85 (70.2%)
	With a mood stabilizer	31 (25.6%)
	Mood stabilizer only	2 (1.7%)
	No pharmacotherapy	3 (2.5%)

Data are presented as mean ± standard deviation (SD), median with first and third quartiles of Me (Q1; Q3), min-max range or count and percentage n (%). Percentages were calculated relative to the entire study group (N = 121).

### 2.2 Study inclusion and exclusion criteria

The study inclusion criteria included the following factors:

- Diagnosis of a mental disorder
- Stable mental condition enabling participation in the study
- Status of a patient treated in judicial detention (preventive measure).
- Maintained ability to understand the content of the study and answer questions
- Expressing informed consent to participate in the study
- Typical drug therapy on the ward

The following factors were listed in the study exclusion criteria:

- Acute psychotic episode, manic episode or severe depression with psychotic symptoms.
- High intensity of negative symptoms preventing cooperation.
- Current suicidal thoughts or self-harming behavior requiring intervention.
- Cognitive deficits
- Inability to obtain informed consent.

Exclusion criteria were based on ethical considerations (the need to obtain informed consent), patient safety (current suicide risk), and measurement reliability (the AIS requires the ability to reflect and understand questions). Individuals with acute exacerbation of symptoms were excluded, including severe psychotic symptoms, significant disorganized thinking, severe negative symptoms and limited communication, severe mood disorders with psychotic symptoms, significant cognitive deficits, and current self-harming behavior or high suicidal risk.

Initial screening of potential participants was performed by the department’s medical staff (psychiatrist and nursing staff), who assessed inclusion and exclusion criteria, particularly current mental status and clinical stability. The role of the treatment team was limited to assessing medical eligibility.

Information about the study and informed consent were obtained by a member of the research team not involved in the patient’s treatment or the preparation of forensic psychiatric opinions. The ability to provide informed consent was assessed individually by a psychiatrist, taking into account the patient’s ability to understand the information, logically consider alternatives, and make a stable and voluntary decision. In case of doubt, the patient was not considered for the study.

To reduce the risk of coercion, the roles of those responsible for treatment and obtaining research consent were separated. Patients were informed of the voluntary nature of their participation, the purpose, and the nature of the study, and were given the opportunity to ask questions in a confidential setting and to withdraw consent at any time, without giving a reason or facing consequences.

### 2.3 Research tools

The study used:

1. Clinical and demographic data questionnaire - analysis of clinical data included two variables collected in a complete manner: the length of the current psychiatric hospitalization and the pharmacotherapy used, in particular the scope and type of antipsychotic therapy. One of the clinical variables for which there were missing data was psychiatric diagnosis. Therefore, the analysis of complete cases (complete case analysis) was applied only to this variable. The remaining analyses were conducted taking into account the actual sample size used in the individual statistical tests. Among the demographic data, age and gender of patients were included in the analyses.
2. The Satisfaction with Life Scale (SWLS) assesses the overall level of life satisfaction. The study used the Polish adaptation by Zygfryd Juczyński (1). The construct validity of the Polish adaptation was verified based on the correlations of SWLS scores with self-esteem, self-efficacy, dispositional optimism, and perceived stress, as well as the results of factor analysis (1). The scale consists of five statements rated on a seven-point scale (1-7). Total scores range from 5 to 35 points, with higher scores indicating greater life satisfaction. Internal consistency of the scale was assessed using Cronbach’s alpha. In the study sample, α = 0.855 (N = 121) was obtained.
3. The Acceptance of Illness Scale (AIS) is used to assess the degree of illness acceptance. The scale was developed by Barbara J. Felton, Tracey A. Revenson, and GA Hinrichsen, and its Polish adaptation by Zygfryd Juczyński was used in the study [27]. Data regarding the psychometric properties of the Polish adaptation also include an assessment of the AIS’s validity. The scale’s validity was assessed, among other things, by comparing its results with physicians’ assessments of treatment outcomes in oncology patients; a significant correlation was obtained (r = 0.42; p < 0.01) [29]. Official materials of the Psychological Testing Laboratory also indicate that the AIS does not have norms, therefore the score ranges used in this study were used solely as a descriptive classification [29]. The scale consists of eight statements rated on a five-point scale (1-5). The total score ranges from 8 to 40 points, with higher values indicating greater illness acceptance. For descriptive classification of the results, the following scale was used: 8–18 points – low acceptance, 19–29 points – medium acceptance, and 30–40 points – high acceptance [28]. This scale was used solely to characterize the study group. The AIS summary score was used to analyze the relationship between illness acceptance and life satisfaction. The internal consistency of the scale was assessed using Cronbach’s alpha. In the study sample, α = 0.845 (N = 121) was obtained.

### 2.4 Statistical analysis

Data were prepared and organized in Microsoft Excel, and statistical analyses were conducted using Statistica 13 (TIBCO Software Inc., 2017). The analysis included 121 patients hospitalized in forensic psychiatry units. Raw summary scores from the Satisfaction with Life Scale (SWLS) and the Acceptance of Illness Scale (AIS) were used in the inferential analyses. The SWLS score ranged from 5 to 35 points, and the AIS score from 8 to 40 points, with higher scores on both scales indicating higher levels of life satisfaction and greater acceptance of illness, respectively.

In the first stage, a descriptive analysis of the SWLS and AIS scores and basic sociodemographic and clinical characteristics of the study group was conducted. Quantitative variables were described using the mean, standard deviation, median, quartiles, and minimum and maximum values. Qualitative variables were presented using counts and percentages. The normality of distribution of quantitative variables and SWLS scores in the compared groups was assessed using the Shapiro -Wilk and Lilliefors tests. In parametric analyses, homogeneity of variance was assessed using Levene’s test. The choice of statistical methods depended on the type of variable and the fulfillment of the assumptions specific to each test.

The relationship between age and SWLS score was assessed using Spearman’s rank correlation coefficient. In this analysis, age was treated as a continuous variable, without any categorical division. Spearman’s rho coefficient was used to determine the direction and strength of the relationship between age and the level of life satisfaction.

Gender differences in SWLS scores were assessed using the Student’s t-test for independent samples. Results were presented as mean and standard deviation. The magnitude of the difference between women and men was assessed using Hedges’ g coefficient. along with a 95% confidence interval.

Differences in SWLS scores by psychiatric diagnosis were assessed using the Student’s t-test for independent samples. The analysis included 116 patients for whom information regarding diagnosis was available. Patients were divided into two groups: those diagnosed with paranoid schizophrenia (n = 80) and those with other psychiatric diagnoses (n = 36). Five patients with missing information regarding diagnosis were excluded from this analysis. Missing information about diagnosis did not exclude these patients from the remaining analyses if the required data were available. For the psychiatric diagnosis variable, a complete case analysis was used, without imputation of missing data.

With respect to pharmacotherapy, two groups were distinguished: patients receiving antipsychotics without a mood stabilizer (n = 85) and patients treated with antipsychotics in combination with a mood stabilizer (n = 31). Those receiving mood stabilizers alone (n = 2) and those not receiving pharmacotherapy (n = 3) were excluded from the comparison. Due to the very small sample size, these patients were not analyzed as separate groups. Comparison of SWLS scores between the two pharmacotherapy groups was performed using the Mann-Whitney U test. Results were presented as median, first, and third quartiles, and effect size was expressed using the r coefficient.

Differences in SWLS scores by length of stay were analyzed after combining the original five length-of-stay categories into three groups: less than 1 year, 1 to 3 years, and more than 3 years. The three longest categories were combined due to their small sample sizes. Differences in SWLS scores between the three groups were assessed using one-way analysis of variance (ANOVA). The effect size for the one-way analysis of variance was determined using the eta-squared (η²) coefficient.

The relationship between illness acceptance and life satisfaction was also analyzed. The relationship between the raw AIS and SWLS summary scores was assessed using Spearman’s rank correlation coefficient. For Spearman’s rank correlation coefficients, 95% confidence intervals were determined using the non-parametric BCa bootstrap method (bias-corrected and accelerated), using 1,000,000 replications. In each replication, complete pairs of observations were randomized with replacement, and Spearman’s rank correlation coefficient was then recalculated.

Student’s t-test, Mann-Whitney U test, and correlation coefficient significance tests were two-sided. A nominal significance level of α = 0.05 was used. No correction was applied to p-values due to multiple testing. P-values were presented to three decimal places.

The study received a positive opinion from the Bioethics Committee of the Medical University of Silesia in Katowice, BNW/NWN/0052KB/94/25. The study was conducted in accordance with the principles of the Declaration of Helsinki and applicable legal regulations regarding research involving hospitalized individuals.

## 3 Results

The results of the statistical analyses are presented in four sections. The first presents the level of life satisfaction assessed using the Satisfaction with Life Scale (SWLS). The second examines the relationship between age and SWLS scores and compares SWLS scores across gender, length of hospitalization, psychiatric diagnosis, and pharmacotherapy regimen. The third presents characteristics of the Acceptance of Illness Scale (AIS) scores. The fourth analyzes the relationship between AIS scores and SWLS scores.

### 3.1 Level of satisfaction with life of hospitalized patients

The level of life satisfaction was assessed using the Satisfaction with Life Scale (SWLS). The scale comprises five statements rated on a seven-point scale (1-7), with the total score ranging from 5 to 35 points, with higher values indicating greater life satisfaction. Table C-13 of the Polish SWLS provisional norms, developed on a group of 555 individuals aged 20-55, representing various levels of education and background, was used to convert raw scores to sten scores (1). Scores of 5–9 points correspond to 1 sten, 10–11 points to 2 sten, 12–14 points to 3 sten, 15–17 points to 4 sten, 18–20 points to 5 sten, 21–23 points to 6 sten, 24–26 points to 7 sten, 27–28 points to 8 sten, 29–30 points to 9 sten, and 31–35 points to 10 sten. Scores of 1–4 sten were interpreted as low, 5–6 sten as average, and 7–10 sten as high (1). The categories 1–4 sten, 5–6 sten, and 7–10 sten corresponded to raw scores of 5-17, 18-23, and 24–35 points, respectively (1). Data are presented in **Tables 2**–**4**.

**Table 2:** Descriptive statistics of raw scores on the Satisfaction with Life Scale (N = 121).

Variable	Descriptive statistics					
	N	M	Me	Minimum	Maximum	SD
SWLS_total	121	19.82	19	5	35	7.50

**Table 3:** Descriptive statistics of sten scores on the Satisfaction with Life Scale (N = 121).

Variable	Descriptive statistics					
	N	M	Me	Min	Max	SD
SWLS_Sten	121	5.31	5	1	10	2.61

**Table 4:** Distribution of life satisfaction levels (N = 121).

SWLS_level	N	%
low	48	39.7%
average	32	26.4%
high	41	33.9%

SWLS raw scores ranged from 5 to 35 points. The mean life satisfaction score in the study group was M = 19.82 (SD = 7.50), and the median Me = 19. The results indicated variation in the level of life satisfaction within the study group.

After converting the raw scores to a sten scale, values ranging from 1 to 10 sten were obtained. The mean sten value was M = 5.31 (SD = 2.61), and the median Me = 5. Both the mean and median were within the range of average sten scores.

Low life satisfaction was found in 48 patients (39.7%), average in 32 (26.4%), and high in 41 participants (33.9%). The largest category consisted of individuals with low life satisfaction, but the numbers in each category were relatively similar.

2. Analysis of the relationship between selected demographic and clinical characteristics and the level of life satisfaction (SWLS)

### 3.2 The relationship between age and the satisfaction with life scale score

The relationship between the age of the participants and the SWLS score was assessed using Spearman’s rank correlation coefficient. No statistically significant relationship was found between the analyzed variables (Spearman’s ρ = -0.166; 95% CI: -0.338 to 0.019; p = 0.068; N = 121). The coefficient value indicated a weak negative correlation.

Detailed results of the correlation analysis are presented in **Table 5**.

**Table 5:** Results of the correlation analysis between age and SWLS score.

Pair of variables	N	p Spearmana	95% CI for Spearman’s ρ	p
Age and SWLS score	121	-0.166	[-0.338; 0.019]	0.068

CI, confidence interval; Spearman’s ρ, Spearman’s rank correlation coefficient.

### 3.3 Comparison of the satisfaction with life scale results according to gender

SWLS scores between women and men were compared using the Student’s t-test for independent samples. The mean SWLS score was slightly higher in the women group than in the men group. It was 20.92 ± 6.92 points among women and 19.53 ± 7.65 points among men.

There was no statistically significant difference in SWLS scores between women and men (t = 0.824; df = 119; p = 0.412). The magnitude of the difference between groups, expressed by Hedges’ g coefficient, was small (g = 0.18; 95% CI: -0.25; 0.62). The results of the comparison are presented in **Table 6**.

**Table 6:** SWLS comparison results between women and men.

Variable	Women (n = 25), mean ± SD	Men (n = 96), mean ± SD	t	df	p	g Hedges (95% CI)
SWLS result	20.92 ± 6.92	19.53 ± 7.65	0.824	119	0.412	0.18 (-0.25; 0.62)

SD, standard deviation; t, Student’s t-test statistic; df, degrees of freedom; CI, confidence interval.

### 3.4 Comparison of the satisfaction with life scale results depending on the psychiatric diagnosis

The analysis included 116 patients with available information on psychiatric diagnosis. Comparison of SWLS scores between patients diagnosed with paranoid schizophrenia and those with other diagnoses was performed using the Student’s t-test for independent samples. SWLS scores in both groups were very similar. Patients diagnosed with paranoid schizophrenia scored an average of 19.79 ± 7.91 points, while patients with other diagnoses scored 19.69 ± 6.82 points. No statistically significant difference was found between the compared groups (t = 0.061; df = 114; p = 0.951).

The magnitude of the difference, expressed by Hedges’ g coefficient, was negligible (g = 0.01; 95% CI: -0.38, 0.40). The results of the comparison are presented in **Table 7**, while the mean SWLS scores along with standard errors and 95% confidence intervals for the means are presented in **Figure 1**.

**Table 7:** SWLS comparison results by psychiatric diagnosis.

Variable	Paranoid schizophrenia (n = 80), mean ± SD	Other recognition (n = 36), mean ± SD	t	df	p	g Hedges (95% CI)
SWLS result	19.79 ± 7.91	19.69 ± 6.82	0.061	114	0.951	0.01 (-0.38; 0.40)

SD, standard deviation; t, Student’s t-test statistic; df, degrees of freedom; CI, confidence interval.

**Figure 1:**
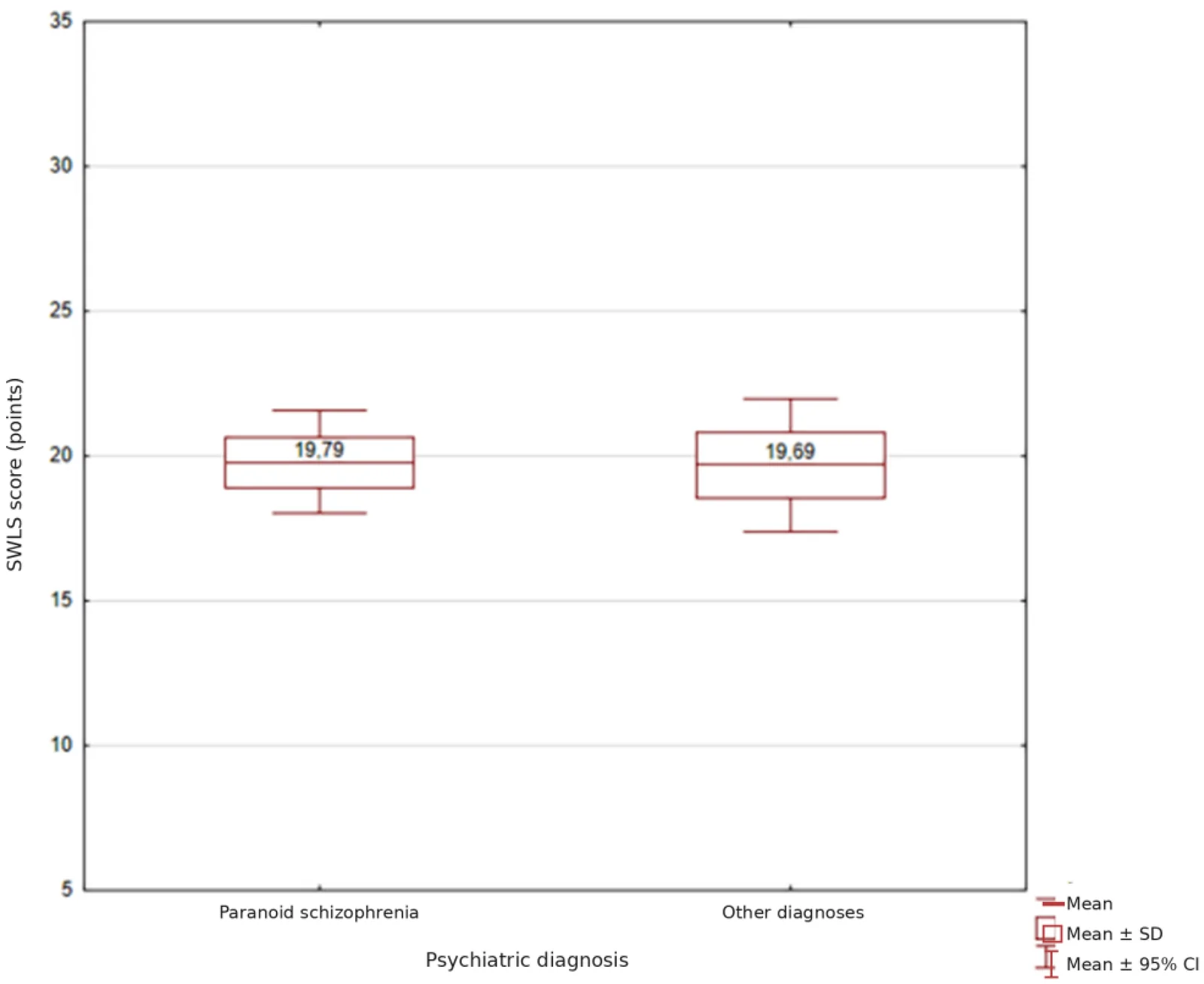
Mean scores on the Satisfaction with Life Scale (SWLS) in groups distinguished by psychiatric diagnosis.

### 3.5 Comparison of the satisfaction with life scale results depending on the pharmacotherapy regimen

A total of 116 patients were enrolled for comparison of pharmacotherapy regimens. In the antipsychotic group, the median SWLS score was 19 points (Q1 = 14; Q3 = 25), whereas in the antipsychotic plus mood stabilizer group, it was 20 points (Q1 = 11; Q3 = 27). Comparison performed using the Mann-Whitney U test revealed no statistically significant difference between the groups (U = 1303; Z = -0.087; p = 0.930). The r coefficient indicated a negligible effect size (r = 0.01). Detailed results of the comparison are presented in **Table 8**, and the distribution of SWLS scores in both groups is presented in **Figure 2**.

**Table 8:** Results of SWLS comparison according to drug therapy regimen.

Variable	Without mood stabilizer (n = 85), Me (Q1; Q3)	With mood stabilizer (n = 31), Me (Q1; Q3)	U	Z	p	r
SWLS result	19 (14; 25)	20 (11; 27)	1303.5	-0.09	0.930	0.01

Me, median; Q1, first quartile; Q3, third quartile; U, Mann-Whitney U test statistic; Z, Z statistic with continuity correction; r, measure of effect size.

**Figure 2:**
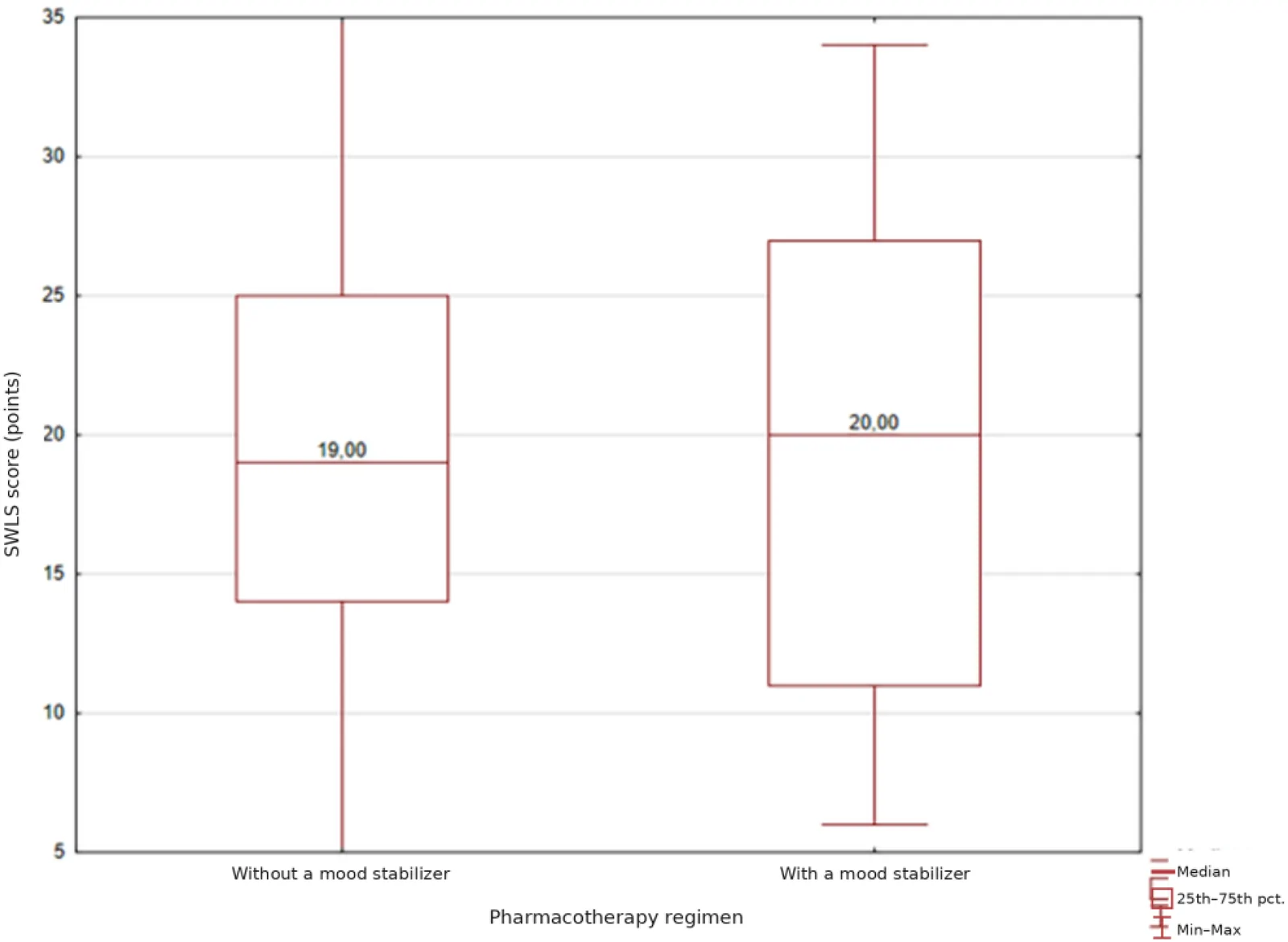
Distribution of Satisfaction with Life Scale (SWLS) results in groups separated according to the pharmacotherapy regimen.

### 3.6 Comparison of the satisfaction with life scale results depending on the length of psychiatric hospitalization

For the purpose of the analysis, the initial classification of hospitalization duration into five categories was reduced to three groups: less than one year (n = 52), 1 to 3 years (n = 36), and more than 3 years (n = 33). The three longest-duration categories were combined due to their small sample sizes.

A comparison of SWLS scores across the three groups was performed using a one-way analysis of variance (ANOVA). The highest mean SWLS score was observed in the group hospitalized for 1 to 3 years (M = 21.67), followed by the group hospitalized for less than one year (M = 19.10), while the lowest score was found in the group hospitalized for more than 3 years (M = 18.94). The one-way ANOVA revealed no statistically significant differences between the groups (F = 1.58; df1 = 2; df2 = 118; p = 0.211; η² = 0.026). Thus, no significant variation in SWLS scores was found among the groups categorized by the duration of psychiatric hospitalization. Detailed results are presented in **Table 9**.

**Table 9:** Results of one-way analysis of variance comparing SWLS scores in three groups of hospitalization time.

Variable	Duration of hospitalization	n	M	SD	Variance analysis				
					df (Effect)	df (Error)	F	p	η²
SWLS_total	Less than a year	52	19.10	7.55	2	118	1,580	0.211	0.026
	From 1 to 3 years	36	21.67	7.36					
	Over 3 years	33	18.94	7.43					

M, mean; SD, standard deviation; df, degrees of freedom; F, F-test statistic; p, p-value; η² eta-squared, a measure of effect size.

## 4 Acceptance of illness level

To further characterize the study group, the results of the Acceptance of Illness Scale (AIS) were also presented. The AIS total score ranges from 8 to 40 points, with higher values indicating a higher level of illness acceptance. The descriptive classification of the results was based on low (8–18 points), medium (19–29 points), and high (30–40 points) levels of acceptance [28].

In the analyzed group of patients, the mean AIS score was M = 28.06 (SD = 7.70), and the median Me = 28. The scores ranged from 8 to 40 points. Detailed descriptive statistics are presented in **Table 10**.

**Table 10:** Descriptive statistics of the overall scores on the Illness Acceptance Scale (N = 121).

Variable	Descriptive statistics					
	N	M	Me	Min	Max	SD
AIS_total	121	28.06	28	8	40	7.70

After classifying the results into three levels of illness acceptance, low levels were found in 13 patients (10.7%), medium levels in 57 (47.1%), and high levels in 51 participants (42.1%). The largest category consisted of individuals with medium levels of illness acceptance. The distribution of illness acceptance levels is presented in **Table 11**.

**Table 11:** Distribution of disease acceptance levels in the study group (N = 121).

AIS_Level	N	%
low	13	10.7%
medium	57	47.1%
high	51	42.1%

### 4.1 The relationship between the level of illness acceptance and the level of life satisfaction

The relationship between the Acceptance of Illness Scale (AIS) and the Satisfaction with Life Scale (SWLS) scores was assessed using the Spearman rank correlation coefficient. A weak positive correlation was found between the scores on both scales, which met the nominal criterion of statistical significance (Spearman’s ρ = 0.215; CI: from 0.023 to 0.387; p = 0.018; N = 121).

Higher life satisfaction scores were accompanied by higher illness acceptance scores. Due to the cross-sectional nature of the study, this result does not allow for determining the direction of the relationship or inferring a causal relationship. The data are presented in **Table 12** and **Figure 3**.

**Table 12:** Correlation between the Satisfaction with Life Scale (SWLS) score and the Acceptance of Illness Scale (AIS) score - Spearman’s rank correlation.

A pair of variables	N	Spearman’s ρ	95% CI for Spearman’s ρ	p
SWLS_total & AIS_total	121	0.215	[0,023; 0,387]	0.018

CI, confidence interval; Spearman’s ρ, Spearman’s rank correlation coefficient.

**Figure 3:**
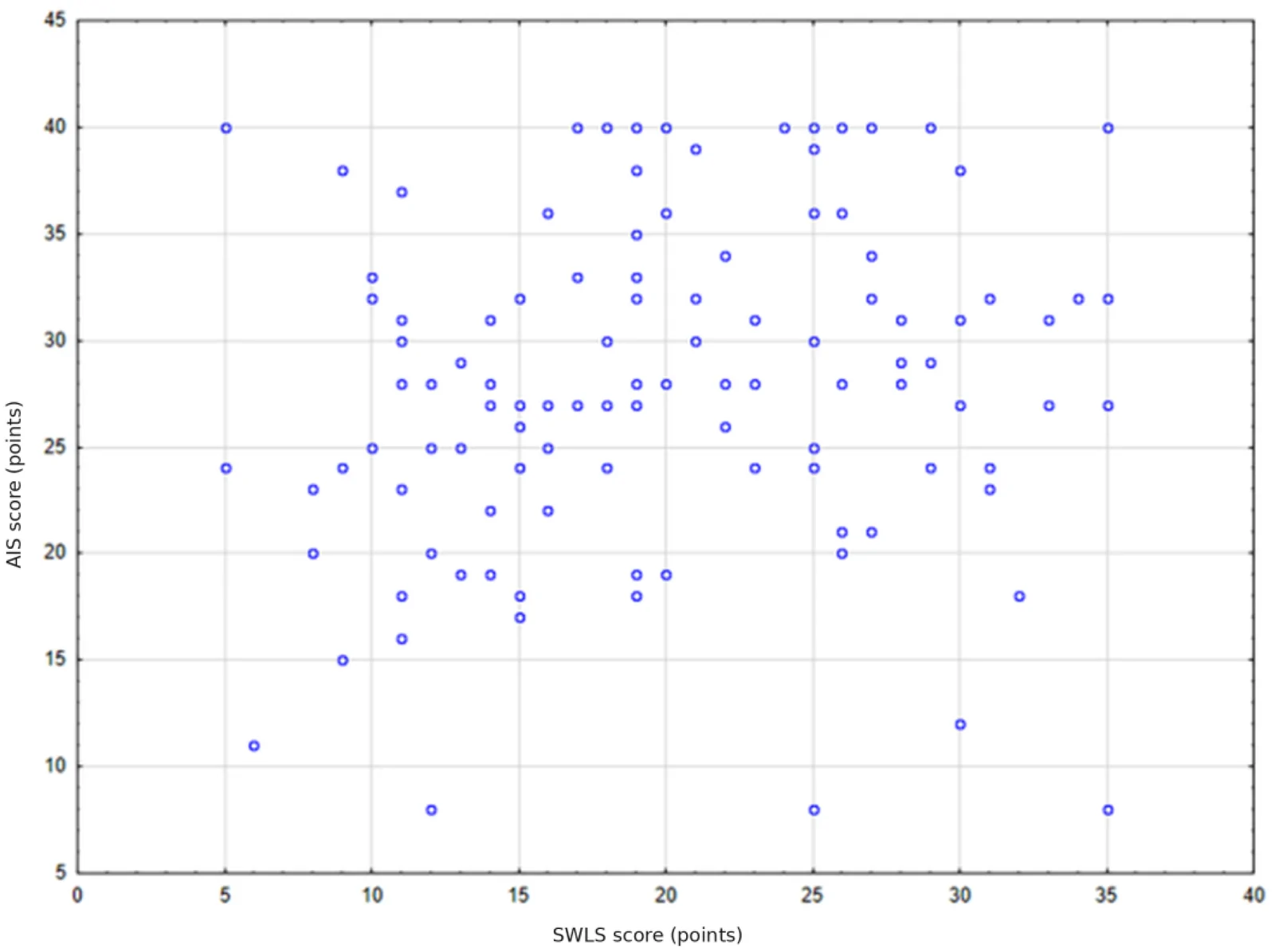
Scatterplot showing the relationship between the Satisfaction with Life Scale (SWLS) score and the Acceptance of Illness Scale (AIS) score.

## 5 Discussion

Life satisfaction is an important dimension of subjective well-being and can provide information that cannot be obtained solely from assessments of psychopathological symptoms or clinical functioning. In the case of forensic psychiatric patients, this indicator seems particularly important due to the co-occurrence of mental illness, long-term hospitalization, limited autonomy, and specific legal conditions.

In the studied group of forensic psychiatry patients, varying levels of life satisfaction were observed. Low satisfaction was found in 39.7% of the study participants, average in 26.4%, and high in 33.9% of patients.

The presence of a relatively large group of patients with low life satisfaction is consistent with previous reports indicating that individuals with severe and chronic mental disorders are characterized by lower levels of subjective well-being than the general population (14–16). At the same time, the specific nature of forensic psychiatry may further influence the perception of one’s life situation. Limited autonomy, compulsory treatment, and uncertainty about the future may constitute additional burdens, regardless of the current severity of psychopathological symptoms (7, 17). Therefore, the low level of satisfaction observed in some participants may be related not only to mental illness but also to the specific nature of functioning in an institutional environment.

The analysis comparing the three groups based on length of hospitalization revealed no statistically significant differences in SWLS scores (p = 0.211). This means that, based on the adopted division of hospitalization length, there was no significant difference in life satisfaction levels between the analyzed groups.

This result is consistent with reports indicating that the length of stay is not necessarily a simple determinant of the subjective quality of life of forensic psychiatric patients (18). It is therefore possible that what matters is not so much the length of hospitalization, but the way in which the patient experiences it.

This interpretation is supported by research indicating the importance of the quality of the therapeutic environment. O’Flynn et al. demonstrated that significant predictors of quality of life in forensic psychiatric patients included, among others, the ability to engage in meaningful activities, the level of security, and the therapeutic nature of the ward environment (21). Of particular interest is the fact that engagement in activities perceived as meaningful by the patient was one of the most important factors related to quality of life. These results suggest that in forensic psychiatric settings, subjective assessment of one’s own life may be more related to the ability to pursue roles, interests, and activities important to the patient than to the duration of stay itself.

Interpersonal relationships and the social climate of the ward may be of similar importance. Bressington et al. demonstrated a relationship between the perceived social climate, the therapeutic relationship, and patient satisfaction with forensic psychiatric services (19). This suggests that a patient’s experience may be shaped by whether they feel treated with respect, listened to, and included in the treatment process. Consequently, even patients staying in the ward for a similar period may demonstrate significantly different levels of life satisfaction, depending on the quality of the relationship and the individual experience of their stay.

Also noteworthy is the lack of significant differences in life satisfaction levels by gender. Although women had a slightly higher mean SWLS score than men (20.92 ± 6.92 vs. 19.53 ± 7.65), this difference was not statistically significant (p = 0.412), and the effect size was small (g = 0.18). Similarly, no difference was found between patients diagnosed with paranoid schizophrenia and those with other psychiatric diagnoses (19.79 ± 7.91 vs. 19.69 ± 6.82; p = 0.951; g = 0.01).

In the study sample, no statistically significant differences in SWLS results were found based on gender or the adopted division of psychiatric diagnosis.

Similarly, no statistically significant difference was found in SWLS scores between the analyzed groups based on pharmacotherapy regimen. The median SWLS score was 19 points in patients treated with antipsychotics without a mood stabilizer and 20 points in patients receiving combined treatment with a mood stabilizer. The difference was statistically insignificant (p = 0.930), and the effect size was negligible (r = 0.01). However, this result should not be interpreted as evidence that pharmacological treatment is irrelevant to patient well-being. The analyzed pharmacotherapy regimen was merely a simplified indicator of treatment type and did not take into account, among other factors, drug doses, duration of use, therapeutic response, adverse events, or symptom severity.

In this study, a weak positive correlation was found between the AIS score and the SWLS score (Spearman’s p = 0.215; p = 0.018). This result met the nominal criterion of statistical significance, but the low strength of the correlation indicates a limited degree of covariation of the analyzed variables. Therefore, the obtained result does not provide a sufficient basis for concluding about the clinical significance of this relationship.

The correlation analysis performed did not take into account other variables at the same time, therefore it does not allow for an assessment of whether the observed relationship would persist after taking them into account.

The low correlation coefficient indicates that the strength of the observed relationship between AIS and SWLS scores was weak. Due to the cross-sectional nature of the study, this relationship should not be interpreted in causal terms, nor should inferences be made about its direction.

The influence of other factors cannot be excluded, such as social support, sense of agency, hope, quality of therapeutic relationships or opportunities to undertake meaningful activities (10, 20, 21).

The obtained results should therefore be considered as a premise for further research on the correlation between illness acceptance and life satisfaction in the forensic psychiatric patient population. Future analyses would be warranted to simultaneously consider other variables and employ multivariate models, which would allow for a more precise assessment of the nature and strength of the observed relationship.

In this context, it is interesting that some of the participants, despite functioning in forensic psychiatry, demonstrated high levels of life satisfaction. This heterogeneity is consistent with previous observations indicating the importance of personal resources, social support, and recovery processes in shaping the well-being of people with severe mental disorders (10, 20). This may indicate that the limitations resulting from forensic hospitalization do not affect all patients in the same way, and their significance is modified by individual resources and the way they interpret their situation.

Autonomy and the ability to participate in decisions regarding one’s own life may also play an important role. Vorstenbosch et al. draw attention to the specific needs of forensic psychiatric patients and the importance of quality of life in the context of the reformed care system, pointing to the need to consider the patient’s perspective and needs in the organization of care (22). In conditions of limited freedom, even limited opportunities to make choices can be important for the sense of subjectivity and control over one’s own life.

The obtained results also have practical implications. Because no statistically significant differences or correlations were found in SWLS scores with respect to the analyzed demographic and clinical characteristics in the study sample, and the level of life satisfaction varied, it seems reasonable to explore modifiable psychosocial and environmental factors. Of particular importance may be the quality of the therapeutic relationship, the social climate of the ward, the ability to engage in meaningful activities, the sense of autonomy, and the patient’s participation in treatment planning (19, 21–23). This study did not directly assess the quality of the therapeutic relationship, the sense of autonomy, the social climate of the ward, or the ability to engage in meaningful activities. These factors, identified in the literature as potentially important for the quality of life of forensic psychiatric patients, may constitute an important area for further research. However, their relative importance compared to the analyzed demographic and clinical variables cannot be determined based on the obtained results. Future research should include direct measurement of these factors and an assessment of their independent and joint relationships with life satisfaction.

Taking into account the patient’s perspective is consistent with the recovery-oriented approach, in which the goal of treatment is not only to reduce symptoms but also to improve functioning and quality of life (20, 26).

Therefore, assessing life satisfaction can be a valuable complement to traditional clinical assessment. Standardized self-report tools allow for the patient’s perspective to be taken into account and can provide information useful in planning individual therapeutic goals (23–25). This is particularly important for forensic psychiatric patients, as improved functioning does not always have to be reflected solely in reduced symptom severity.

### 5.1 Limitations of the study

When interpreting the obtained results, several limitations of this study should be considered. First, its cross-sectional nature prevents conclusions about causal relationships between the analyzed variables, allowing only the identification of co-occurring relationships. Second, the size of the study group, particularly with respect to some categories of analyzed variables, may have limited the statistical power of the analyses and thus the ability to detect small effects. This limitation primarily concerned the group of women and selected categories of pharmacotherapy used, which should be taken into account when interpreting the obtained results.

Third, the study was conducted among patients meeting specific eligibility criteria, excluding those with acute mental health conditions or significant cognitive impairments. Although justified by methodological considerations and the ability to obtain reliable data, the adopted criteria limit the generalizability of the results to the entire population of forensic psychiatric patients, including those with more severe psychopathological symptoms and more profound cognitive deficits.

Fourth, the range of clinical variables considered did not encompass all potential factors that may be related to life satisfaction. In particular, failure to consider the full spectrum of psychological, social, and environmental conditions may have limited the possibility of a comprehensive interpretation of the studied phenomenon. Another limitation is the lack of p-value correction due to the multiple inferential analyses conducted, which increases the risk of obtaining a nominally significant result due to multiple testing. Therefore, the nominally significant result of the AIS-SWLS correlation should be interpreted with caution and treated as requiring confirmation in further research.

An additional limitation is the interpretation of the results obtained on the Satisfaction with Life Scale (SWLS). The mean raw score in the study group was 19.82 ± 7.50 points, while the mean sten value was 5.31 ± 2.61. Low life satisfaction was found in 39.7% of the participants, average in 26.4%, and high in 33.9%. However, it should be emphasized that this interpretation is based on sten norms, the adequacy of which in relation to the specific population of forensic psychiatry patients requires further verification. Furthermore, the sten norms used to interpret SWLS results were developed for a group of individuals aged 20–55 years, whereas the age of the participants in this study ranged from 19 to 74 years. Therefore, the interpretation of sten scores from individuals outside the age range of the norming group requires particular caution. The obtained results should therefore be treated primarily as a characteristic of the level of life satisfaction in the studied sample, and not as clear evidence of reduced well-being in this clinical population.

Another limitation of the study is the inability to link individual observations to specific centers, which precluded an assessment of inter-center differences and potential within-center clustering of results. Although all centers operated under the same legal regulations governing forensic psychiatry, differences in care organization and the therapeutic environment—which could have influenced patient life satisfaction—cannot be ruled out.

These limitations justify caution in interpreting the results and suggest the need for further research, encompassing larger and more diverse patient groups, taking into account a broader range of clinical, psychological, and social factors. Longitudinal studies would also be important to assess changes in life satisfaction over time and to verify the adequacy of the psychometric tools and norms used in the forensic psychiatric patient population.

It is also worth noting the specificity of the “other psychiatric diagnoses” category, which included patients with diagnostic entities other than paranoid schizophrenia. Due to the heterogeneity of this group and the impossibility of conducting reliable comparisons within individual diagnostic entities, the obtained results should be interpreted with caution. It should be emphasized that aggregating various diagnoses into a single category may have limited the ability to reveal individual-specific differences in life satisfaction. The lack of significant differences between groups does not mean, therefore, that individual diagnoses are characterized by the same level of life satisfaction.

In summary, the results indicate varying levels of life satisfaction within the study group. There was no statistically significant correlation between age and SWLS score, nor were there any significant differences in SWLS scores based on gender, psychiatric diagnosis, pharmacotherapy regimen, or length of hospitalization. However, a weak positive correlation was found between the AIS score and SWLS score, which met the nominal criterion of statistical significance. These results suggest that further research should pay particular attention to psychosocial and environmental factors that may be associated with variations in the well-being of forensic psychiatric patients.

## 6 Conclusions

1. Among the patients included in the study, varying levels of life satisfaction were found. Low life satisfaction was observed in 39.7% of the study participants, average in 26.4%, and high in 33.9%.
2. In the study group, the most common level of disease acceptance was medium (47.1%) or high (42.1%), while the low level of acceptance was observed in 10.7% of the respondents.
3. There was no statistically significant correlation between age and SWLS score, nor any significant differences in SWLS scores due to gender, adopted psychiatric diagnosis, analyzed pharmacotherapy regimen, or length of hospitalization.
4. Of the presented inferential analyses, only the correlation between the AIS and SWLS scores met the nominal criterion of statistical significance. The correlation was positive and weak, meaning that higher illness acceptance scores were accompanied by higher life satisfaction scores. Due to the low strength of the correlation, this result should not be automatically considered clinically significant. Due to the cross-sectional nature of the study, it is not possible to draw conclusions about a causal relationship or its direction.
5. The obtained results indicate the need for further investigation of psychosocial and environmental factors that may be associated with life satisfaction in forensic psychiatric patients. Their importance, including therapeutic relationships, autonomy, sense of agency, and the ability to participate in meaningful activities, should be verified in future studies using direct measurements of these variables.

## Data Availability

The original contributions presented in the study are included in the article/supplementary material. Further inquiries can be directed to the corresponding author.
